# When Do We Really Need Coronary Calcium Scoring Prior to Contrast-Enhanced Coronary Computed Tomography Angiography? Analysis by Age, Gender and Coronary Risk Factors

**DOI:** 10.1371/journal.pone.0092396

**Published:** 2014-04-08

**Authors:** Gitsios Gitsioudis, Waldemar Hosch, Johannes Iwan, Andreas Voss, Edem Atsiatorme, Nina P. Hofmann, Sebastian J. Buss, Stefan Siebert, Hans-Ulrich Kauczor, Evangelos Giannitsis, Hugo A. Katus, Grigorios Korosoglou

**Affiliations:** 1 University of Heidelberg, Department of Cardiology, Heidelberg, Germany; 2 University of Heidelberg, Department of Diagnostic and Interventional Radiology, Heidelberg, Germany; 3 University of Heidelberg, Institute of Psychology, Heidelberg, Germany; University of Groningen, Netherlands

## Abstract

**Aims:**

To investigate the value of coronary calcium scoring (CCS) as a filter scan prior to coronary computed tomography angiography (CCTA).

**Methods and Results:**

Between February 2008 and April 2011, 732 consecutive patients underwent clinically indicated CCTA. During this ‘control phase’, CCS was performed in all patients. In patients with CCS≥800, CCTA was not performed. During a subsequent ‘CCTA phase’ (May 2011–May 2012) another 200 consecutive patients underwent CCTA, and CCS was performed only in patients with increased probability for severe calcification according to age, gender and atherogenic risk factors. In patients where CCS was not performed, calcium scoring was performed in contrast-enhanced CCTA images. Significant associations were noted between CCS and age (r = 0.30, p<0.001) and coronary risk factors (χ^2^ = 37.9; HR = 2.2; 95%CI = 1.7–2.9, p<0.001). Based on these associations, a ≤3% pre-test probability for CCS≥800 was observed for males <61 yrs. and females <79 yrs. According to these criteria, CCS was not performed in 106 of 200 (53%) patients during the ‘CCTA phase’, including 47 (42%) males and 59 (67%) females. This resulted in absolute radiation saving of ∼1 mSv in 75% of patients younger than 60 yrs. Of 106 patients where CCS was not performed, estimated calcium scoring was indeed <800 in 101 (95%) cases. Non-diagnostic image quality due to calcification was similar between the ‘control phase’ and the ‘CCTA’ group (0.25% versus 0.40%, p = NS).

**Conclusion:**

The value of CCS as a filter for identification of a high calcium score is limited in younger patients with intermediate risk profile. Omitting CCS in such patients can contribute to further dose reduction with cardiac CT studies.

## Introduction

Recent technical developments with coronary computed tomography angiography (CCTA) constituted an important step forward for the non-invasive diagnostic work-up of symptomatic patients with suspected or known coronary artery disease (CAD) [1]. However, CCTA is still affected by numerous limitations, including blooming artefacts mainly caused by coronary calcification. This may account for a higher rate of false positive and false negative results [2], [3], even in the era of modern 256 or 320-slice CT scanners [4]. Therefore, routinely used CCTA protocols generally incorporate a ‘filter scan’ for the assessment of Coronary Calcium Scoring (CCS), in order to identify patients with severe coronary calcification, where the usefulness of CCTA for CAD detection is considered uncertain by current guidelines [5].

Radiation exposure represents the major limitation of CCS and CCTA, since both are associated with a non-negligible risk for cancer [6], [7]. However, with current dose reduction strategies (dose modulation, prospective ECG-gating, low-tube voltage imaging and iterative reconstruction algorithms) the radiation exposure for CCTA can be substantially reduced, so that meanwhile the relative dose for CCS may equal or even be higher than that required for CCTA [8]–[10]. From this point of view, and in light of the limited prognostic and diagnostic value of CCS in symptomatic patients [11]–[13], its usefulness as a filter scan prior to CCTA needs to be reconsidered.

In the present study we therefore investigated the contribution of CCS and CCTA to the total radiation exposure using different acquisition protocols. During our ‘*control phase*’ we tested the ability of clinical parameters (age, gender and atherogenic risk profile) to differentiate between patients with heavily calcified vessels versus those where severe calcification is unlikely, so that CCS could be avoided in this context. During the subsequent *‘CCTA phase’* we then verified the ability of this algorithm to avoid CCS prior to CCTA and the extent of the resultant radiation savings.

## Methods

### Patient Population

During the ‘*control phase*’ consecutive patients who underwent CCTA for suspected or known coronary artery disease (CAD) between February 2008 and April 2011using 64-slice (n = 130) or 256-slice CT scanners (n = 602) were prospectively analyzed in terms of clinical characteristics imaging parameters, and resultant radiation exposure. All these patients underwent CCS and CCTA.

During the subsequent *‘CCTA phase’* between May 2011 and May 2012 another 200 consecutive patients underwent 256-slice CCTA. CCS was performed only in patients with increased pretest-probability for heavily calcified vessels according to their age, gender and atherogenic risk factors.

Patient body weight, height and body mass index (BMI), and traditional CAD risk factors, including 1) advanced age (>65 yrs.), 2) arterial hypertension (blood pressure≥140/90 mmHg or antihypertensive therapy), 3) hyperlipidemia (triglycerides≥190 mg/dL, LDL-cholesterin≥115 mg/dL or antilipidemic treatment), 4) cigarette smoking (self-reported), 5) diabetes mellitus (HbA1c>6.5% or antidiabetic treatment) and 6) a family history of CAD (self-reported) were recorded at the time of the CCTA. Based on the sum of these risk factors a score was built (range 0–6) and the Duke Clinical Score, which incorporates type of chest discomfort, age, gender, and traditional atherogenic risk factors [14] was calculated for each patient. Furthermore, cardiac medications, laboratory parameters including serum creatinine, urea, total cholesterol, low-density lipoprotein cholesterol (LDL-C), high-density lipoprotein cholesterol (HDL-C) and serum triglycerides were acquired. All procedures complied with the Declaration of Helsinki, were approved by our local ethic committee of the University of Heidelberg (S317/2008) and all patients gave written informed consent.

### CT imaging procedures

#### Patient Preparation

Patient preparation included the intravenous administration of incremental doses of 2.5 mg of metoprolol (range 0–30 mg), (Novartis, Pharma GmbH) 20–30 min before the CT scan in patients with heart rates ≥60 beats/min. In addition, glyceryl nitrate (800 micrograms sublingual spray) was administrated immediately before the CT scan for coronary vasodilatation.

#### Acquisition Protocols and Imaging Parameters

**Figure 1:**
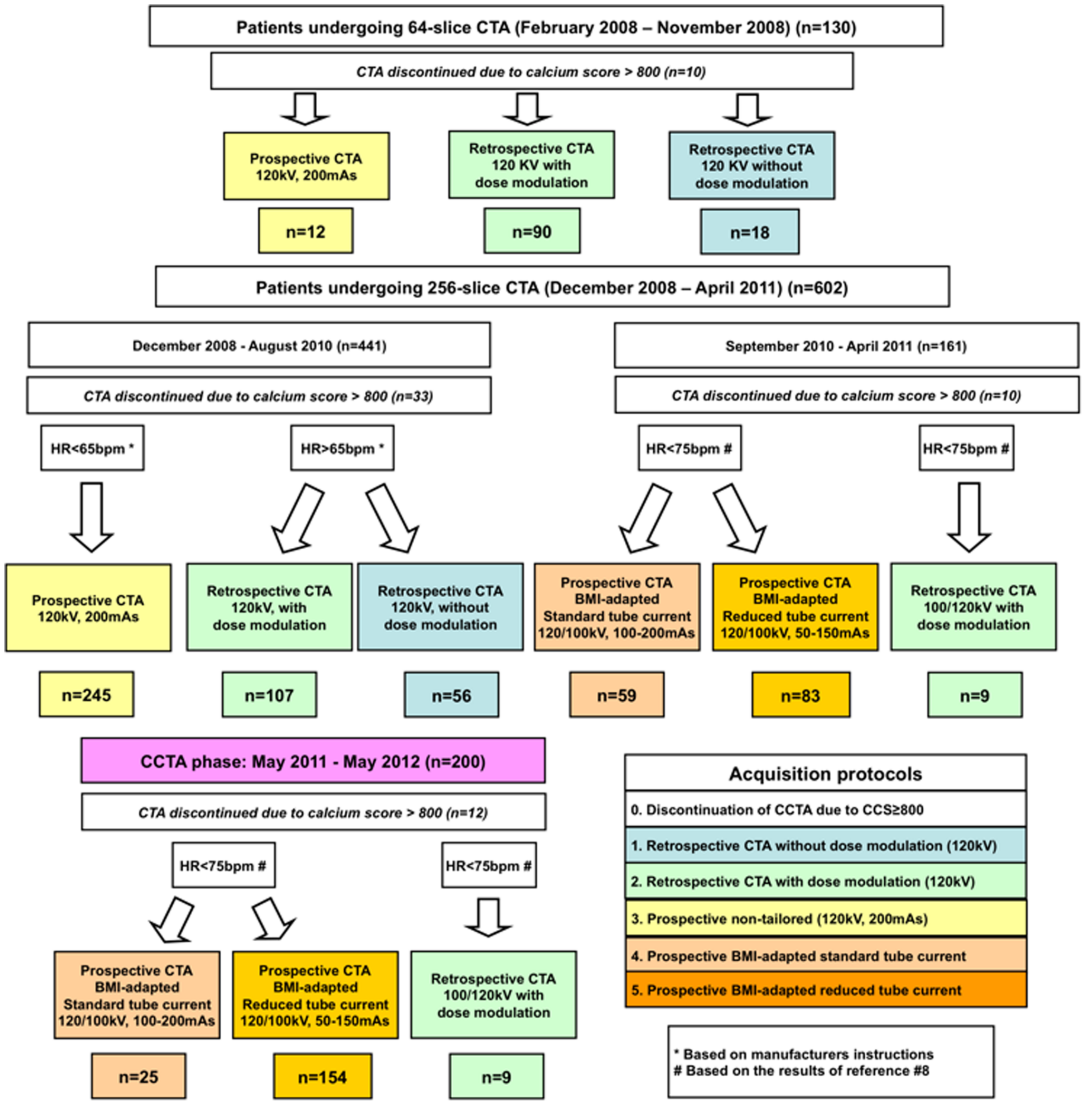
*Flow chart.* 732 consecutive ‘control phase’ and 200 ‘CCTA phase’ patients undergoing 64-slice or 256-scile CCTA and using different acquisition protocols.

From February until November 2008 scans were performed using a 64-slice scanner (Philips Healthcare, Cleveland, Ohio) and since December 2008 using a 256-slice Brilliance iCT scanner (Philips Healthcare, Cleveland, Ohio). Scans were performed ECG-gated either retrospectively or prospectively (‘Step & Shoot Cardiac’) depending on the patient's heart rate and using either 120 kV or 100 kV depending on patients body mass index (BMI) and the availability of the 100 kV tube (available in our institution since September 2010; overview presented in Figure 1).

#### Coronary Calcium Score (CCS)

CCS was performed in all patients of our ‘control phase’ and in a selected group of patients in our ‘CCTA cohort’ using the following imaging parameters: tube voltage of 120 kV with an effective tube current-time product of 55 mAs per section, slice collimation 32×0.625-mm acquisition and a 0.33 s gantry rotation time. The resultant images were used for coronary calcium quantification using the Agatston Score method and dedicated application software (Philips Extended Brilliance Workspace 4.5, Cleveland, Ohio, US). In patients with a predefined cut-off of CCS≥800, indicative of severe coronary calcification, CCTA was not performed.

In patients during the CCTA phase, where CCS was *not* performed, based on criteria determined during our ‘control phase’, CCS was estimated on CCTA images using an algorithm previously described by Bischoff et al. [15] (Figure S1). In order to validate this algorithm in our cohort, we firstly tested this approach in 145 randomly selected cases where both CCS and CCTA images where available, demonstrating a high correlation between the 2 techniques (r = 0.97, p<0.001). Bland Altman plots demonstrated no trend for systematic over- or underestimation for CCS estimation using CCTA images (Figure S2). Furthermore, CCS was estimated using CCTA images in all 200 patients of the CCTA cohort, who did not undergo CCS scans.

#### Coronary Computed Tomography Angiography (CCTA)

For CCTA a bolus of 80 ml of contrast agent (Ultravist 370, Bayer Schering Pharma) was injected intravenously using an antecubital line (18GA, BD Venflon TM Pro Safetly), as described previously [8]. The scan started automatically using a bolus tracking with a region of interest placed in the descending aorta and a threshold of 110 HU. The entire volume of the heart was acquired during one breath-hold in 4–7 s with simultaneous ECG recordings.

#### Retrospectively ECG-gated CCTA

Retrospectively ECG-gated CCTA was performed either with or without tube current modulation using the following imaging parameters: tube voltage of 120 kV with an effective tube current-time product of 800–1050 mAs per slice, slice collimation 64×0.625 mm acquisition and a 0.27 s gantry rotation time with the 256-slice scanner. With the 64-slice scanner on the other hand, slice collimation was 64×0.625-mm with a gantry rotation time of 0.40 s and tube current-time product of 600–900 mAs per slice.

#### Prospectively ECG-triggered CCTA

For prospectively ECG-triggered CCTA (‘Step & Shoot Cardiac’) the starting point was defined in the early end-diastolic phase at 75% of the RR interval. The detector configuration was 2×128×0.625 mm, with 256 overlapping slices of 0.625 mm thickness and dynamic z-focal spot. Acquisitions were performed using either 100 or 120 kV tube voltage and with an effective tube current-time product of 50–200 mAs per slice for the 256-slice and 150–210 mAs for the 64-slice Brilliance CT scanner, depending on patients BMI.

In our study the following prospectively triggered CCTA protocols have been performed: (1) protocol with fixed (non-tailored) tube current (120 kV and 200 mAs), (2) BMI-adapted protocol with standard tube current (100 or 120 kV and 100–200 mAs), and (3) BMI-adapted protocol with reduced tube current (100 or 120 kV and 50–150 mAs) (see Figure 1 for details).

### Assessment of Image Quality

Image quality was assessed in all patients semi-quantitatively by 2 experienced readers (W.H. and G.K.) in consensus, according to the 15-segment coronary artery model of the American Heart Association and on the basis of the presence of motion artifacts or coronary calcification:

*1 = diagnostic image quality, i.e. no visible effects or mild to moderate effects of motion or calcification without degradation of image quality,*

*2 = non-diagnostic image quality due to severe motion artifacts and*

*3 = non-diagnostic image quality due to severe calcification.*

### Estimation of the Radiation Dosage

The dose-length product (DLP) was obtained from the patient protocol of the system. The effective dose was calculated for all scans, based on DLP and an organ weighting factor for the chest at the investigated anatomic region (k = 0.014 mSv×(mGy×cm)^−1^) averaged between male and female models [8], [10], [16].

### Statistical Analysis

Analysis was performed using commercially available software (MedCalc software, Mariakerke, Belgium). Continuous variables are presented as mean±standard deviation unless otherwise indicated. Differences in radiation exposure between different CCTA acquisition protocols were compared using ANOVA with Bonferroni adjustment for multiple comparisons. Group differences between ordinal variables were tested using the exact Mann-Whitney test, and differences between nominal variables were assessed using Fisher exact tests. All tests were 2-tailed. Correlations between calcium scoring and age or biochemical markers were performed using linear regression analysis. Based on the association of CCS with age, gender and atherogenic risk factors, the ability of such parameters was investigated to predict CCS≥800 and CCS≥400 using receiver operating characteristics (ROC) analysis. Cut-off values were selected for age (in subgroups by gender and coronary risk factors), in order to predict the presence of CCS≥800 and CCS≥400 with a negative predictive value of >97% (i.e. ≤3% pre-test probability for CCS≥800 and CCS≥400). ROC curve analysis was performed using the methodology described by DeLong et al. [17]. Inter- and intra-observer variability for CCS assessment was obtained by repeated analysis of 40 representative cases. Differences were considered statistically significant at p<0.05.

## Results

### Demographic Data

Clinical and laboratory data of our control and CCTA cohort are illustrated in Table 1. Clinical and imaging parameters were in most cases similar between the 2 cohorts. Patients of the CCTA cohort were slightly younger, and had a slightly lower number of coronary risk factors, but exhibited similar pre-test probability for CAD. Significant differences on the other hand, were noted in terms of age, CAD risk factors, biochemical markers and clinical presentation in patients with CCS≥versus<800 Agatston units.

**Table 1:** Demographic, clinical, laboratory and hemodynamic data.

Parameters	All ‘control phase’ patients (n = 732)	All ‘CCTA phase’ patients (n = 200)	p-Values	Control phase patients with CCS<800 (n = 679)	Control phase patients with CCS≥800 (n = 53)	p-Values
	***Demographics and coronary risk factors***					
*Age (yrs.)*	63±11	61±11	0.02	62±11	72±9	<0.001
*Male sex*	348 (48%)	112 (56%)	NS	314 (46%)	34 (64%)	0.01
*Body mass index (kg/m^2^)*	27.3±4.9	26.9±4.9	NS	27.3±5.0	26.8±4.1	NS
*1. Advanced age (>65 yrs.)*	325 (44%)	75 (38%)	NS	282 (42%)	43 (81%)	<0.001
*2. Arterial hypertension*	579 (79%)	132 (73%)	NS	526 (77%)	53 (100%)	<0.001
*3. Hypercholesterolemia*	481 (66%)	94 (52%)	<0.001	435 (64%)	46 (87%)	<0.01
*4. Diabetes mellitus*	104 (14%)	26 (14%)	NS	88 (13%)	16 (30%)	0.001
*5. Family history of CAD*	242 (33%)	65 (36%)	NS	231 (34%)	11 (21%)	0.03
*6. Smoking*	217 (30%)	50 (28%)	NS	194 (29%)	23 (43%)	0.02
*Number of risk factors (0–6)*	2.7±1.2	2.5±1.3	0.04	2.6±1.2	3.6±0.9	<0.001
*Pre-test probability (%)*	51±31%	48±29%	NS	49±30%	79±20	<0.001
	***Laboratory data***					
*Total cholesterol (mg/dl)*	192±48	195±51	NS	192±48	185±44	NS
*LDL cholesterol (mg/dl)*	112±36	115±37	NS	112±36	108±33	NS
*HDL cholesterol (mg/dl)*	54±18	53±15	NS	55±18	47±11	<0.01
*Serum triglycerides (mg/dl)*	139±86	149±101	NS	134±88	151±58	NS
*Serum creatinine (mg/dl)*	0.89±0.27	0.88±0.24	NS	0.88±0.27	0.98±0.30	0.02
*Serum urea (mg/dl)*	36±14	34±11	NS	36±14	40±15	0.07
	***Cardiac medications***					
*Aspirin (100 mg/day)*	325 (44%)	74 (44%)	NS	294 (43%)	31 (58%)	0.03
*Clopidogrel (75 mg/day)*	62 (8%)	4 (2%)	0.004	50 (7%)	12 (23%)	<0.001
*β blockers*	387 (53%)	80 (48%)	NS	350 (52%)	37 (70%)	0.01
*ACE inhibitors*	202 (28%)	58 (35%)	NS	179 (26%)	23 (43%)	<0.01
*Angiotensin receptor blockers*	191 (26%)	34 (20%)	NS	173 (25%)	18 (34%)	NS
*Diuretics*	201 (27%)	37 (22%)	NS	173 (25%)	28 (53%)	<0.001
*Statins*	344 (47%)	75 (45%)	NS	304 (45%)	39 (74%)	<0.001
*Cumarines*	74 (10%)	17 (10%)	NS	65 (10%)	9 (17%)	0.09
	***Clinical presentation***					
*Typical angina*	40 (6%)	13 (8%)	NS	34 (6%)	6 (13%)	0.09
*Atypical angina*	157 (27%)	50 (30%)	NS	145 (27%)	12 (25%)	NS
*Non-cardiac chest pain*	392 (67%)	101 (62%)	NS	362 (67%)	30 (62%)	NS
*Exertional dyspnoea*	254 (39%)	59 (36%)	NS	229 (41%)	25 (49%)	NS
	***Hemodynamic data prior to imaging***					
*Heart rate (bpm)*	63±10	62±8	NS	64±10	65±12	NS
*Systolic pressure (mmHg)*	133±18	134±19	NS	133±18	138±19	NS
*Diastolic pressure (mmHg)*	80±11	82±12	NS	80±11	80±8	NS
	***Results calcium scoring***					
*Total Calcium Scoring*	212±462	272±652**	NS	110±204	1447±770	<0.001

Data presented as number of patients and percentages or as mean±standard deviation.

LDL indicates Low Density Lipoprotein; HDL, High Density Lipoprotein; CAD, Coronary Artery Disease; NS, Not Significant, and NA, Not Applicable.

**Measured (n = 94) or estimated (n = 106) CCS using non-contrast and CCTA images, respectively.

### Protocols for CCTA, Radiation Exposure and Image Quality

**Figure 2:**
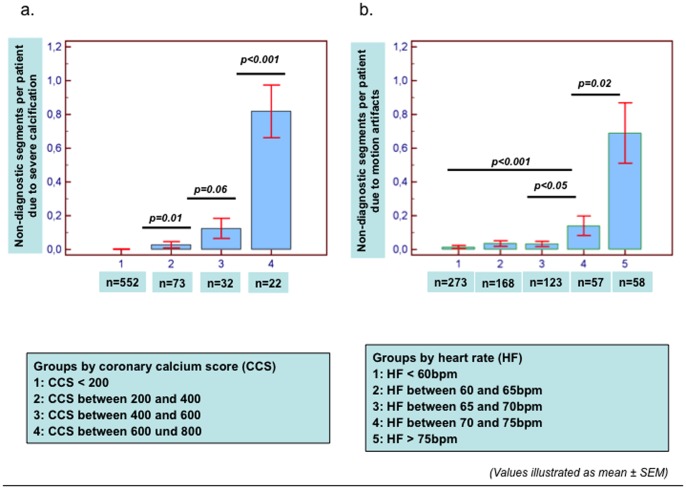
*Non-diagnostic segments with* ‘control phase’ *studies*. The number of non-diagnostic segments due to calcification or due to motion artifacts was associated with increasing total CCS (a) and increasing heart rates (b), respectively. Significant differences in terms of non-diagnostic segments due to calcification and motion artifacts were observed especially with CCS>600 (a) and heart rates>75 bpm (b), respectively.

Overall 87 of 10,185 coronary segments (0.85%) were deemed as non-diagnostic due to motion artifacts (n = 62; 0.61%) or due to severe calcification (n = 25, 0.25%) in our control cohort. The number of non-diagnostic segments due to calcification or due to motion artifacts increased with increasing CCS and heart rate, respectively (Figure 2).

**Figure 3:**
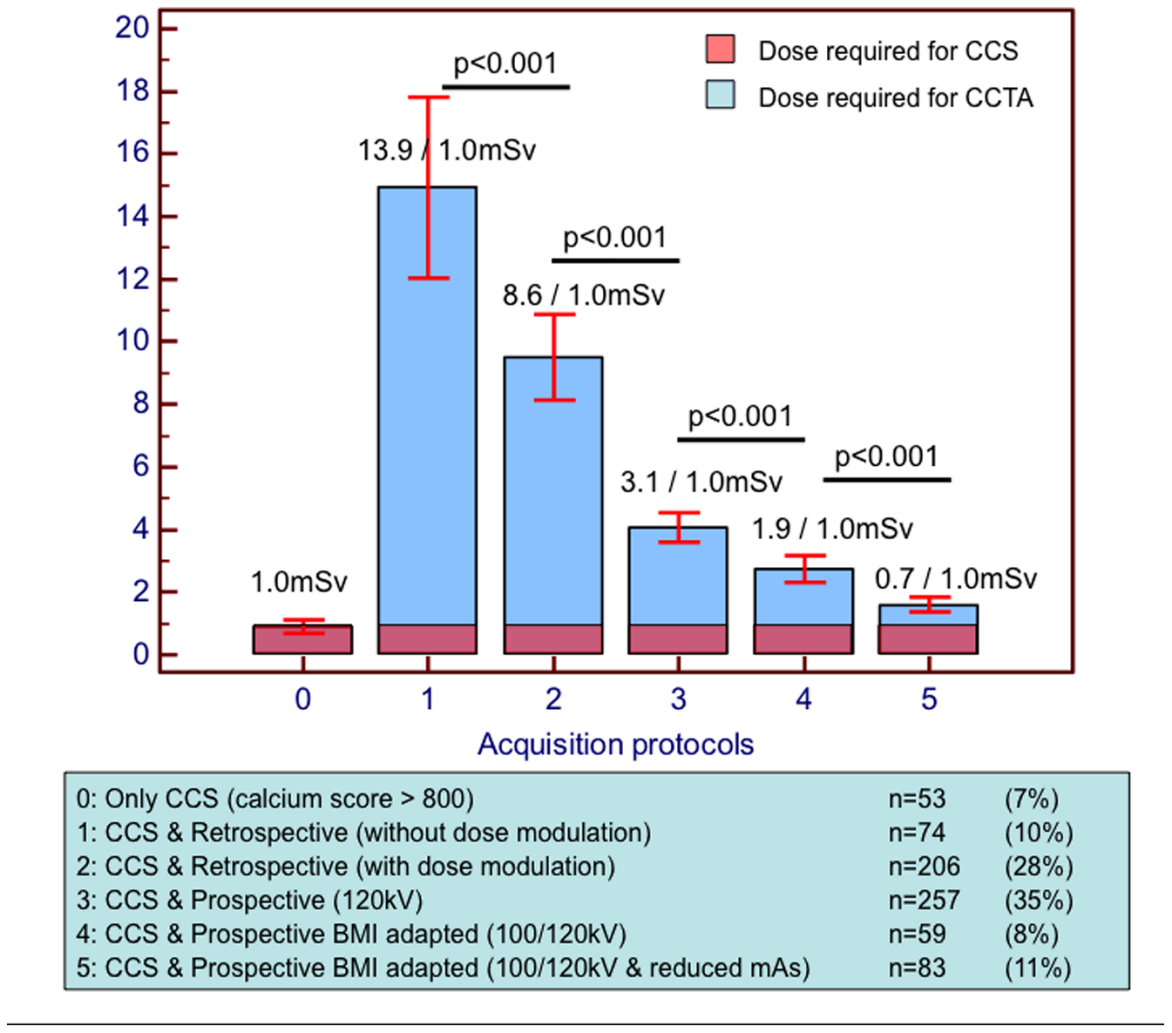
*Contribution of CCS and CCTA to total radiation exposure using prospective versus retrospective CCTA protocols*. With retrospective CCTA, CCS contributed to only 6–9% of the total radiation exposure. Conversely, with prospective scans, CCS contributed to 40–50% of the total radiation exposure, when low-tube voltage and BMI-adapted imaging parameters were applied.

In our control cohort CCTA was not performed only in 53 of 732 (7%) patients due to CCS≥800. The absolute radiation exposure and the relation of CCS to CCTA using different prospective versus retrospective CCTA protocols, is illustrated in Figure 3. Using retrospective CCTA, CCS contributed to only 6–9% of the total radiation exposure. Conversely, with prospective scans, CCS contributed up to ∼50% of the total radiation exposure, depending on the applied protocol.

Radiation exposure due to CCTA, including the rate of pro- versus retrospective scans in the course of time during our study can be appreciated in our Figure S3.

### Prediction of Calcium Score ≥800 by Age and Risk factors

**Figure 4:**
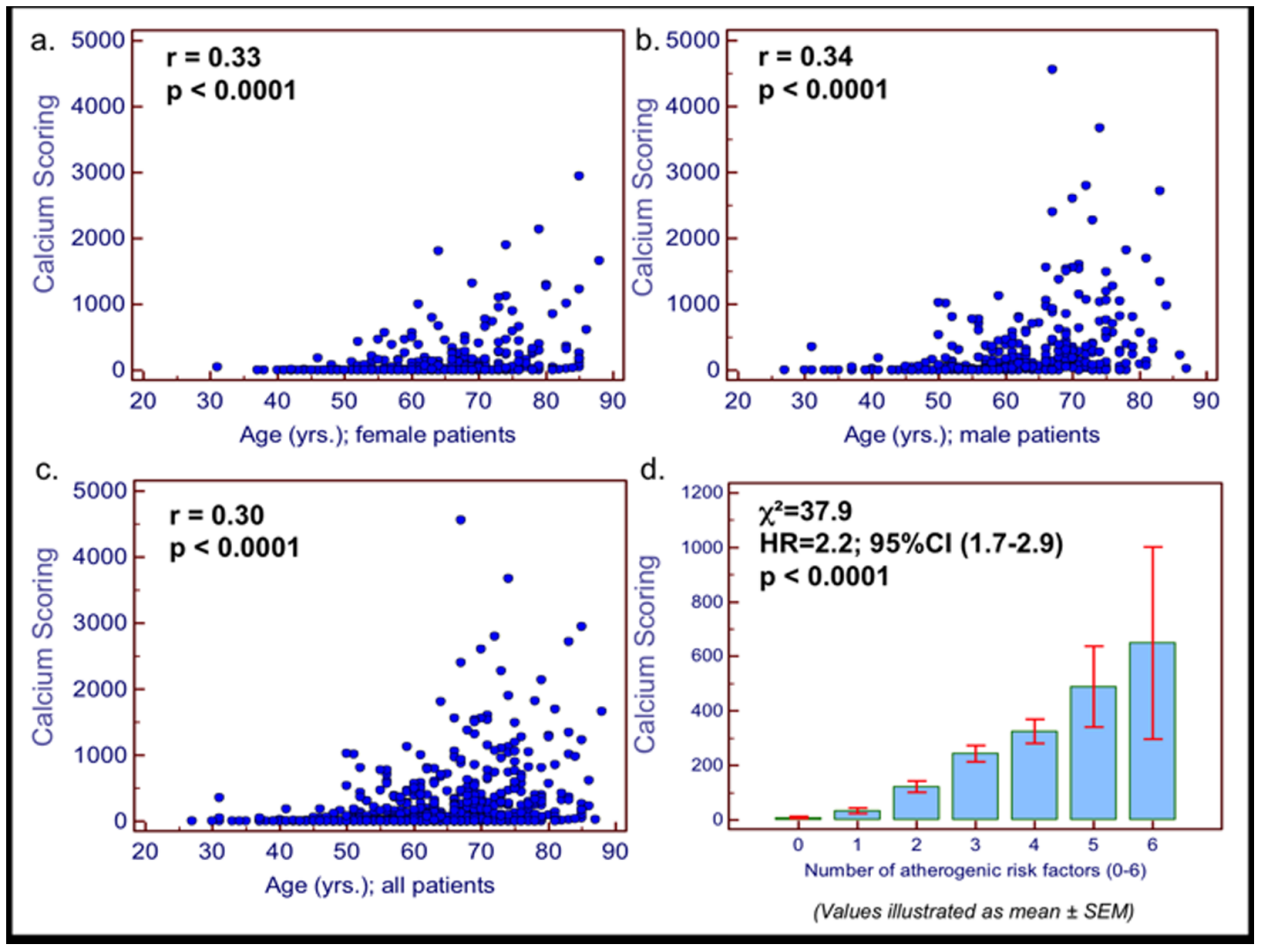
*Correlation between calcium scoring and clinical parameters*. Significant associations were observed between total calcium scoring with age (a–c; in both male and female patients; r = 0.33 for female; r = 0.34 for male and r = 0.30 for all patients, p>0.001 for all) and with the total number of atherogenic risk factors (d; χ^2^ = 37.9; HR = 2.2, 95%CI = 1.7–2.9; p<0.001).

Significant associations were observed between total calcium scoring with age (in both male and female patients (Figure 4a, b, c) and with the total number of atherogenic risk factors (Figure 4d). Using *ROC* analysis, we found that age and atherogenic risk factors are predictive of CCS≥800 (AUC_age_ = 0.77, AUC_risk factors_ = 0.65). This association was present in subgroups including patients with ≤2 risk factors, female and male patients (Figure S4).

Based on our analysis, for male patients <61 yrs. and for female patients <79 yrs. a negative predictive value of >97% was present for CCS≥800 (≤3% pre-test probability for CCS≥800), (Table 2). For CCS≥400 on the other hand, a negative predictive value of >97% was present with lower ages <46 yrs. for male and <55 yrs. for female patients.

**Table 2:** Negative predictive values based on patient age and atherogenic risk factors for the prediction of calcium scoring≥800 or ≥400 prior to CTA based on the 732 ‘control phase’ patients.

	All patients	Patients with ≤2 risk factors	Patients with ≥3 risk factors	Male patients	Female patients
	**Prediction of calcium scoring≥800**				
NPV>95%	73 yrs.	86 yrs.	66 yrs.	67 yrs.	88 yrs.
NPV>97%	67 yrs.	74 yrs.	62 yrs.	61 yrs.	79 yrs.
NPV>99%	50 yrs.	62 yrs.	49 yrs.	50 yrs.	63 yrs.
	**Prediction of calcium scoring≥400**				
NPV>95%	55 yrs.	69 yrs.	48 yrs.	49 yrs.	68 yrs.
NPV>97%	50 yrs.	66 yrs.	45 yrs.	46 yrs.	55 yrs.
NPV>99%	45 yrs.	50 yrs.	45 yrs.	45 yrs.	51 yrs.

Numbers indicate patient's age (in yrs.), NPV indicates negative predictive value.

### CCTA phase

**Figure 5:**
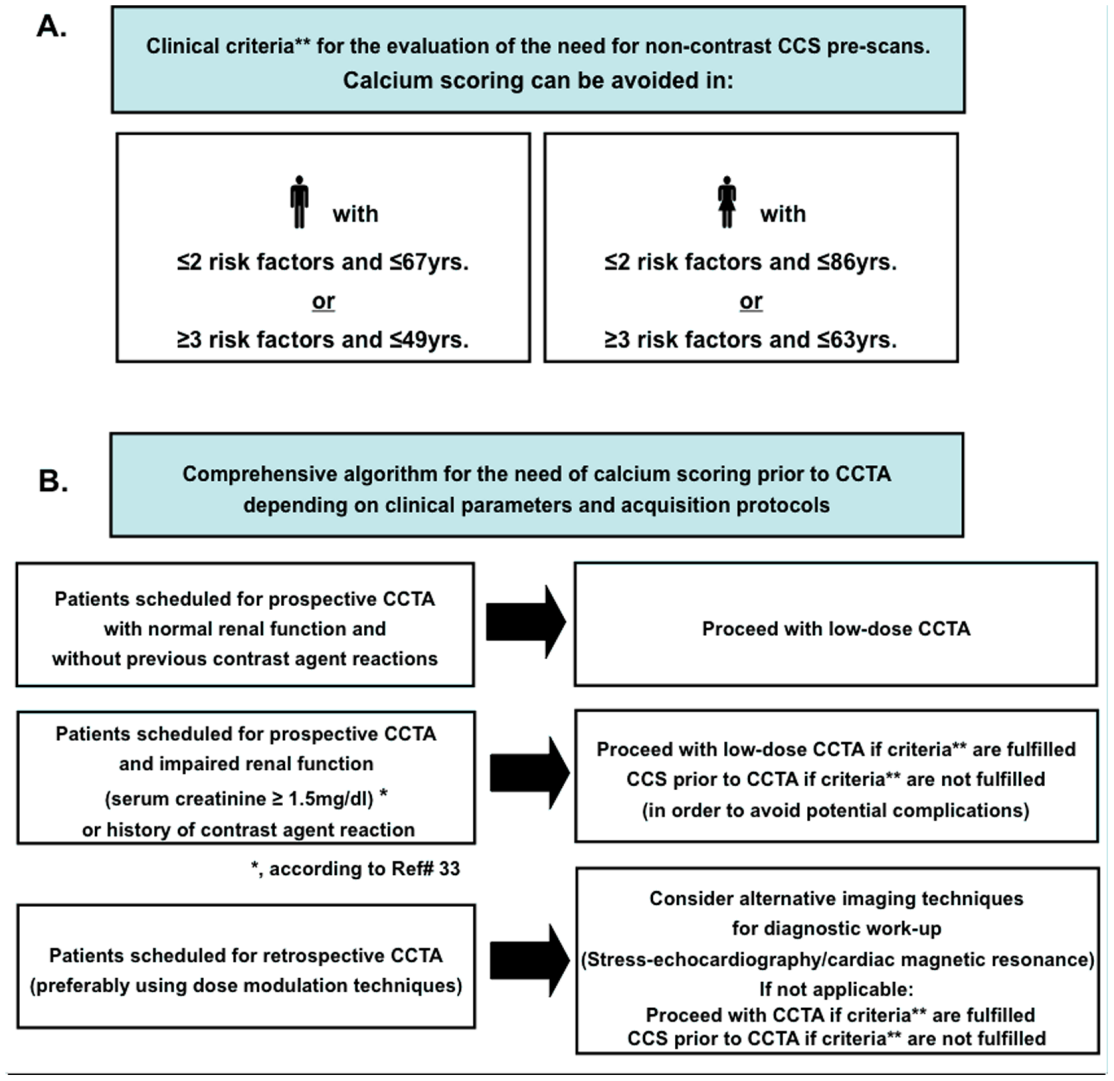
Criteria for identifying patients with low probability for CCS≥800 and implementation in the clinical routine. A. Criteria determined during the control phase for the identification of patients, where CCS does not need to be performed (i.e. patients with ≤3% pre-test probability for CCS≥800). B. Implementation of the proposed algorithm in the clinical routine.

During our CCTA period, and based on the criteria determined during our control phase (Figure 5A), CCS was not performed in 106 of 200 (53%) patients, including 47 of 112 (42%) males and 59 of 88 (67%) females. In patients under 60 yrs., CCS was not performed in 63 of 84 patients (75%), including 34 of 50 (68%) males and 29 of 34 (85%) females).

**Figure 6:**
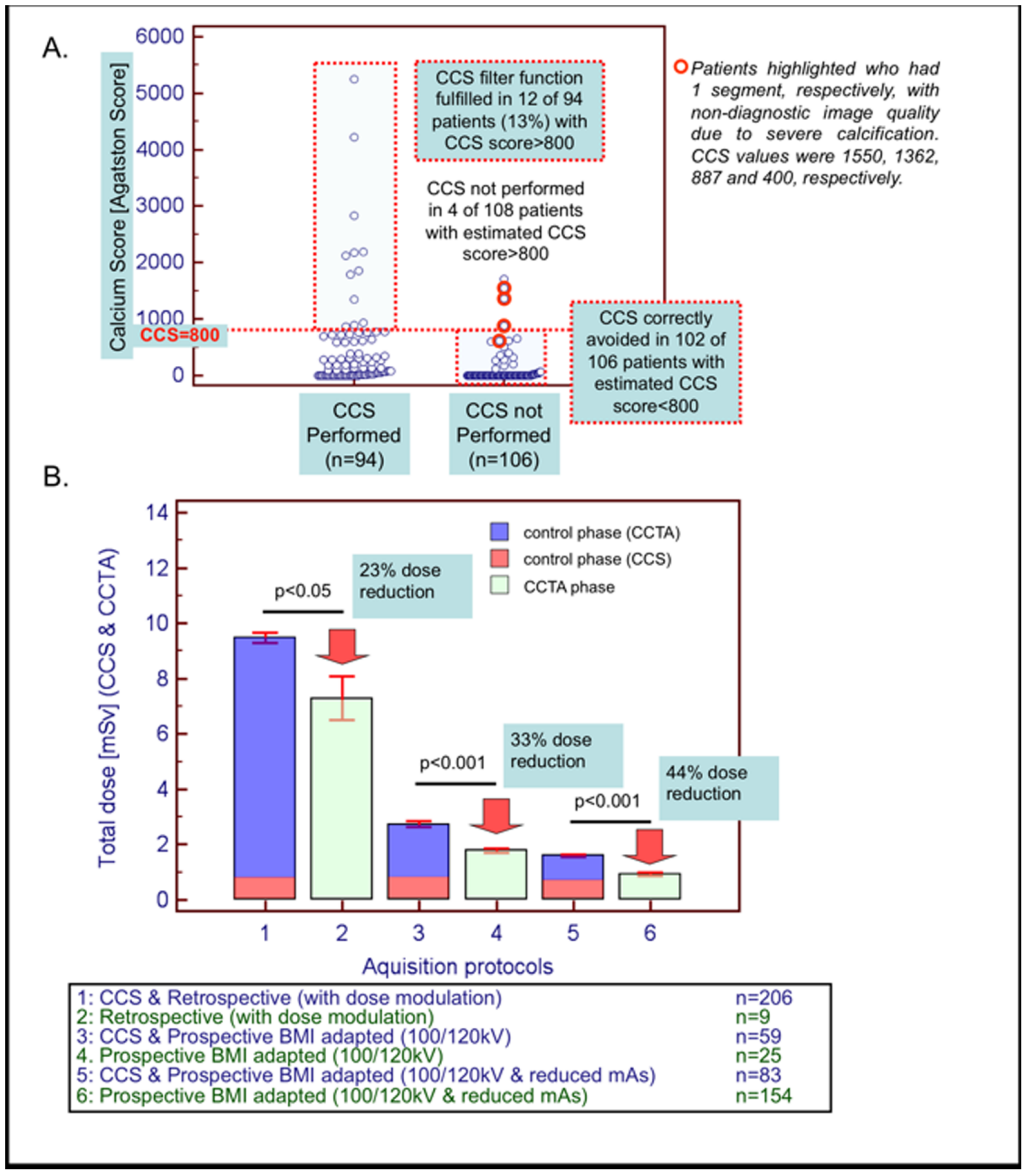
*CCTA phase data*. A. In patients where non-contrast scans were not performed (n = 106 the estimated CCS was significantly lower compared to that in patients who underwent CCS scans (n = 94; p<0.001). In patients where CCS scans were not performed (n = 106), estimated CCS was >800 Agatston units in only 4 of 106 patients, while in the remaining patients estimated CCS was ≤800 Agatston units. B. Total radiation exposure was significantly lower, compared to patients scanned with corresponding CCTA protocols during our ‘control phase’ study (p<0.05 for all protocols used).

In patients who underwent CCS (n = 94), the latter fulfilled its filter function in only 12 patients, who indeed exhibited CCS>800 (mean of 2208±1345 Agatston units). Conversely, in patients where CCS scans were not performed (n = 106), estimated CCS>800 was present in only 4 of 106 patients (3.7%) (Figure 6A).

Non-diagnostic image quality due to calcification was present only in 12 of 2820 (0.40%) coronary segments (corresponding patients highlighted red in Figure 6A), which is similar to that observed during the control phase (0.25% versus 0.40%, p = NS). Non-diagnostic image quality due to motion artefacts was present in 20 of 2820 (0.70%) coronary segments (p = NS compared to 0.60% during the control phase).

In the CCTA group the resultant total radiation exposure was significantly lower, compared to patients scanned with corresponding protocols during the control phase (relative radiation savings of 23%, 33% and 44% in patients undergoing retrospective CCTA with dose modulation, prospective BMI-adapted standard and prospective BMI-adapted reduced tube current CCTA, respectively) (Figure 6B).

### Inter- and intra-observer variability

For the calculation of CCS low inter- and intra-observer variability values of 1.4% and 1.1% were observed, respectively.

## Discussion

This is to our knowledge the first study, which systematically analyzes the value of CCS as a filter prior to clinically indicated CCTA. Based on our results, CCS with a resultant radiation exposure of ∼1 mSv does not need to be performed in the majority of patients scheduled for CCTA, *especially in younger and female patients who have the highest attributable life-time cancer risk* [6]. Using BMI-adapted 100 kV tube voltage prospectively triggered acquisition protocols [18], [19] radiation exposure due to CCS is almost similar or even higher than that required for CCTA, so that the notion not to perform CCS prior to CCTA appears very attractive. This algorithm allows for reducing the radiation exposure with coronary CT studies, while maintaining an accurate cardiovascular risk assessment, because the CCS can still be assessed using CCTA scans, if required [15].

Maintaining sufficient image quality for a reliable diagnosis of CAD with a minimum of radiation exposure is the major challenge with CCTA. Within the last few years, a variety of strategies including tube current dose modulation [8], [20], prospective ECG-triggering [8], [21], [22], low-tube voltage imaging [9] and iterative reconstruction techniques [10] were proposed and tested in order to reduce radiation dose due to CCTA. In our study the mean dose required for CCTA was ∼5 mSv, which is lower than the median dose observed in recent multi-centre trials and similar to that required for X-Ray coronary angiography [23]. This dose gradually reduced over time due to the stepwise implementation of dose reduction strategies during the study period [18], [19], [24], illustrated in our Figure S3. After the implementation of such strategies, CCTA can be obtained with ∼1.0 to 1.5 mSv, so that CCS amounted for ∼40–50% of the total radiation exposure.

In asymptomatic patients and in patients who underwent nuclear ischemia testing, previous studies demonstrated the value of CCS and CCS progression beyond the assessment of established atherogenic risk factors and the delineation of regional ischemia for the prediction of clinical outcomes [25]–[27]. This can be explained by the complementary nature of CCS, a surrogate marker of coronary anatomy and total plaque burden and nuclear scintigraphy, a modality that can detect the functional significance of coronary lesions. In addition, a sub-study from the CONFIRM registry recently demonstrated high negative predictive values of 96% and 99%, respectively for the exclusion of obstructive CAD (50% and 70% stenosis, respectively) in patients with zero CCS [28]. In the same line a zero CCS was associated with an increased net reclassification index compared to conventional atherogenic risk factors in asymptomatic patients [27]. Furthermore, the usefulness of a CCTA-based procedure for the safe discharge of symptomatic patients with zero CCS from emergency department has also been demonstrated [29]. However, the role of CCS for both monitoring and adaption of preventive therapies [30], [31] as well as for the prediction of CAD in symptomatic patients is still controversial [11]–[13], [27], [32]–[34]. In addition, a zero or low calcium score can also be estimated using CCTA images without the need of prior native scans as shown in our study and in previous reports [15].

In the clinical realm, CCS is routinely performed in most centres prior to CCTA in order to identify patients with severe coronary calcification, *where the usefulness of CCTA for CAD detection is uncertain according to current guidelines* [5]. Indeed, even with new generation multi-slice scanners, which can theoretically decrease the problem of calcium blooming due to faster gantry rotation times, Z-direction focal-spot sampling and spherical detector design, stenosis severity may be overestimated in heavily calcified coronary segments [4], [35]–[37]. Therefore, in most previous studies using 64-slice CCTA, patients with CCS≥400 or CCS≥600 were excluded from analysis [3], [38], [39]. In our study and due to potential technical advantages of the 256-slice scanner the cut-off for CCS was set at 800, which is identical to that set in more recent studies [15], [35]. In the same line, in a recent 256-slice CCTA study, the diagnostic accuracy of the techniques was reported to be acceptable between CCS values of 400 and 1000, whereas the number of false positive findings started increasing with values >1000 Agatston Units [4]. For 64-MDCT and newer CT systems on the other hand, high diagnostic sensitivity for CAD detection could be shown even in patients with high coronary calcification [40]. However, specificity still remains low in such patients with severe coronary calcification, whereas further prospective randomized clinical studies with 256- of 320-MDCT scanners are now warranted to clarify the impact of CCS cut-off values on the diagnostic ability of CCTA. In our study, over 99% of the available coronary segments showed diagnostic image quality. However, an increase of non-diagnostic segments was observed with increasing calcification, particularly beyond a calcium score of 600 as shown in Figure 2A. This justifies our a priori selected cut-off value of CCS≥800 as a criterion for discontinuation of CCTA studies in this cohort and underscores the usefulness of CCS as a filter scan prior to CCTA in patients with severe coronary calcification. In this context, it should be noted that the prevalence of CAD significantly influences the importance of image quality for the correct diagnosis or exclusion of CAD. Thus, in cohorts with low prevalence of CAD, exclusion of significant coronary lesions may be easier compared to cohorts with higher extent of calcification and higher prevalence of obstructive CAD. In this regard, the presence of obstructive CAD was relatively low in our cohort, so that our current results cannot be extrapolated in cohorts with increased pre-test probability and higher prevalence of obstructive CAD. However, the low presence of obstructive CAD in our cohort is associated with the fact that cardiac CT was performed primarily in patients with low- or intermediate and not in patients with high pre-test probability for CAD, which is in agreement with current guidelines [41].

Using CCS pre-scans in high-risk patients (impaired renal function, hyperthyroidism) can help to avoid potential nephrotoxic or thyroid related complications due to contrast agent administration during CCTA [42]. In male patients <61 yrs. and female patients <79 yrs., and particularly in those with low risk profile, CCS was found to be unnecessary as a filter scan prior to CCTA. Implementing our results in the clinical workflow, we propose an algorithm for avoiding CCS scans prior to CCTA based on simple clinical parameters, including both patient's age, gender and risk profile and in dependence of the scheduled acquisition protocol (Figure 5B). In our ‘CCTA cohort’, 102 of 106 patients where CCS was not performed had an estimated CCS<800. This indeed resulted in radiation savings of ∼1.0 mSv, which represents up to *40–50%* of the total radiation exposure, when contemporary radiation exposure reduction strategies were applied. Simultaneously, diagnostic image quality was maintained in this cohort and non-diagnostic segments due to calcification were present to a similar extent to that observed during the ‘control phase’. The notion that this additional radiation saving due to CCS can be obtained using very simple algorithms especially in younger and in female patients with increased attributable life-time risk of cancer [43], makes the translation of our findings to the clinical realm promising. Previous studies investigated the radiation induced cancer risk due to CCS scans using radiation risk models [7]. In these studies, the radiation dose from a single CCS scan was found to vary more than 10-fold (effective dose range between 0.8 and 10.5 mSv) depending on the protocol. Taking a median dose of ∼2.3 mSv into account, a CCS scan at the age of 40 yrs. was estimated to result in a radiation induced cancer risk of 9 and 28 cancers per 100,000 men and women, respectively, which is definitely a non-negligible finding. Cancer risk increased with decreasing age at the time of the CCS scan, and with increasing effective dose. Consequently, it becomes clear that the dose due to CCS scans should be reduced by using prospectively ECG triggered protocols or if possible completely avoided, especially in the younger and in females, as anticipated in our study.

### Limitations

Patients were examined with different CT scanners and CCTA acquisition protocols. However, the current study is intended to be an *effectiveness study* (i.e. a study in which referring physicians rather than the study design dictated the selection of patients for imaging and the use of different acquisition protocols). Furthermore, the CCS protocol was identical for all patients, and the cut-off of 800 as criterion for discontinuation of CCTA studies was selected ‘a priori’. Of course, some cardiac CT centers may *empirically* do not perform CCS scans prior to CCTA in younger and especially in female patients with low atherogenic risk profile. However, to our knowledge this is the first study, which systematically investigates this specific issue, and the results provided by our analysis can be easily implemented in the clinical workflow. CCS has been previously reported to aid planning the scan volume of CCTA, resulting dose reduction in some cases [44], [45]. However, the expected dose reduction due to more accurate planning is expected to be substantially lower than that achieved by avoiding pre-scan CCS, especially when low-tube voltage, BMI-adapted prospective CCTA is applied. Indeed, the absolute radiation savings during our CCTA phase approached ∼1.0 mSv, which represents the dose required for CCS. Of course, the clinical benefit of this radiation savings remains unclear and can definitely not be addressed by our study, especially given the present debate, that <10 mSv may have no significant biological mal-effects [46]. Finally, the age and gender specific cut-off values provided for low risk patients, where CCS does not need to be performed prior to CCTA depend on the selection of the parameters considered in such a statistical model. Thus, the selection of a different list of risk factors or of a different cut-off value like 400 Agatston units for CCS would likely yield different results.

### Conclusion

The value of CCS as a filter for identification of a high calcium score is limited in younger patients with intermediate risk profile. Based on easily acquired clinical parameters as age and atherogenic risk factors, CCS as a filter scan prior to CCTA can be avoided in the majority of patients scheduled for CCTA. Thus, although in older patients with more than 2 atherogenic risk factors, CCS can still be considered as a useful filter scan prior to CCTA and to contrast agent administration, in younger and female patients, with the highest *attributable life-time risk for lung and breast cancer*, avoiding CCS may aid together with contemporary radiation reduction strategies for further reduction of radiation exposure. However, the avoidance of CCS in view of the potential value of CCS in symptomatic patients has to be further elucidated.

## Supporting Information

Figure S1 **Examplary calcium scoring in a conventional non-contrast enhanced (A,B) and in a coronary CT angiography scan (C,D) in the same patient.** Standard HU threshold for determination of Agatston score of 130 HU was used in (A,B) whereas for (C,D) a threshold of 456 HU was set depending on the density in the ascending aorta.
(TIFF)

Figure S2 ***Correlation between CCS measured by typical non-contrast scans and estimated using CCTA images*** **.** A high correlation was observed between the 2 measure techniques (r = 0.97, p<0.001) without a trend for systematic over- or underestimation.
(TIF)

Figure S3 ***Radiation exposure and percentage of prospective versus retrospective CCTA in the course of time during our study period*** **.** After the implementation of dose reduction strategies like prospective ECG-triggering, low-tube voltage and BMI-adapted imaging, CCTA can be obtained with ∼1.0 to 1.5 mSv, so that CCS amounts for ∼40–50% of the total radiation exposure.
(TIFF)

Figure S4 ***Receiver operating characteristic analysis*** **.** Age and atherogenic risk factors were predictive of CCS≥800 in patients who underwent CCTA (a). Age was also predictive of CCS≥800 in patients with ≤2 risk factors (b) and both in male (c) and female patients (d).
(TIF)
