# Supplementary figures and images for: When Do We Really Need Coronary Calcium Scoring Prior to Contrast-Enhanced Coronary Computed Tomography Angiography? Analysis by Age, Gender and Coronary Risk Factors
Source: PLoS One. 2014 Apr 8;9(4):e92396. doi: 10.1371/journal.pone.0092396 (PMC3979653; doi:10.1371/journal.pone.0092396)

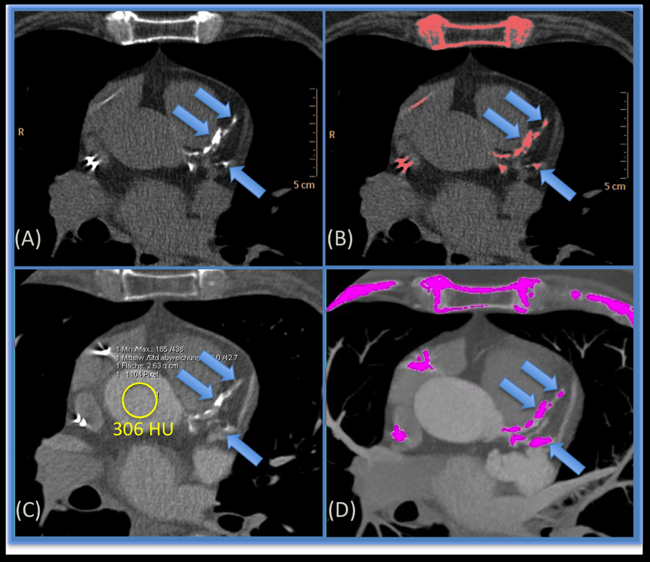

Supplement: Figure S1 — Examplary calcium scoring in a conventional non-contrast enhanced (A,B) and in a coronary CT angiography scan (C,D) in the same patient. Standard HU threshold for determination of Agatston score of 130 HU was used in (A,B) whereas for (C,D) a threshold of 456 HU was set depending on the density in the ascending aorta. (TIFF) [file pone.0092396.s001.tif]

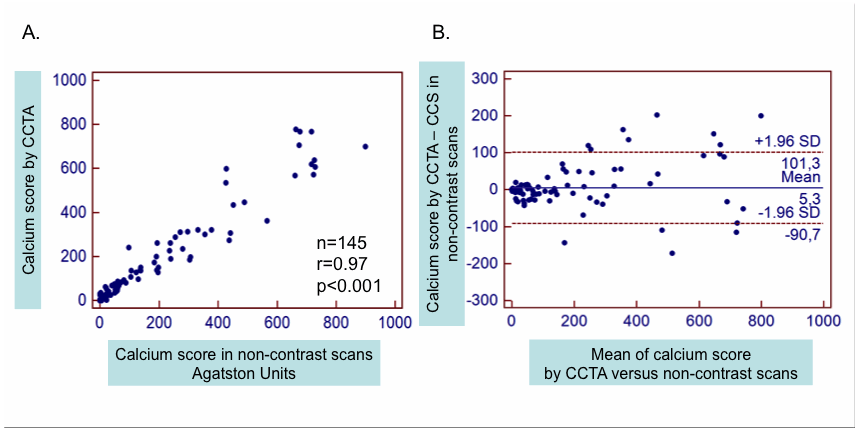

Supplement: Figure S2 — Correlation between CCS measured by typical non-contrast scans and estimated using CCTA images . A high correlation was observed between the 2 measure techniques (r = 0.97, p<0.001) without a trend for systematic over- or underestimation. (TIF) [file pone.0092396.s002.tif]

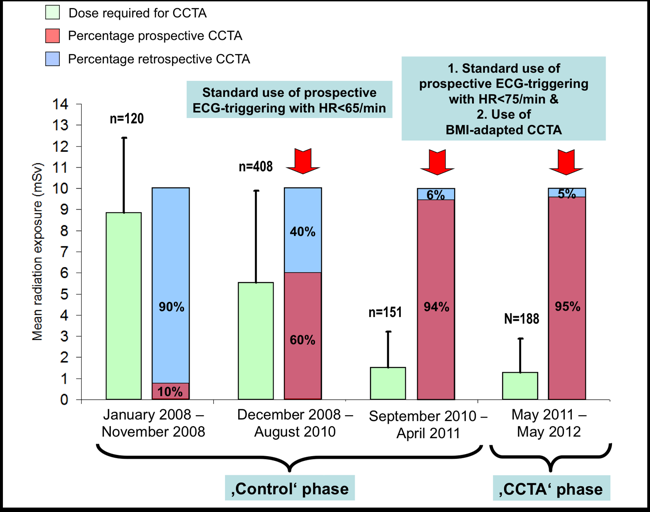

Supplement: Figure S3 — Radiation exposure and percentage of prospective versus retrospective CCTA in the course of time during our study period . After the implementation of dose reduction strategies like prospective ECG-triggering, low-tube voltage and BMI-adapted imaging, CCTA can be obtained with ∼1.0 to 1.5 mSv, so that CCS amounts for ∼40–50% of the total radiation exposure. (TIFF) [file pone.0092396.s003.tiff]

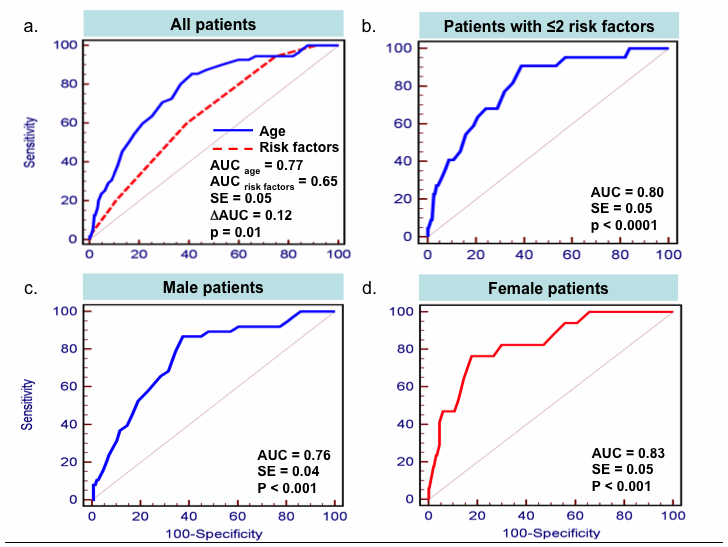

Supplement: Figure S4 — Receiver operating characteristic analysis . Age and atherogenic risk factors were predictive of CCS≥800 in patients who underwent CCTA (a). Age was also predictive of CCS≥800 in patients with ≤2 risk factors (b) and both in male (c) and female patients (d). (TIF) [file pone.0092396.s004.tif]
